# Eating disorder symptoms are prospectively associated with higher BMI percentile in male early adolescents

**DOI:** 10.1007/s40519-026-01824-w

**Published:** 2026-02-16

**Authors:** Jason M. Nagata, Abubakr A. Al-Shoaibi, Shayna Weinstein, Zain Memon, Elizabeth J. Li, Wesley R. Barnhart, Christiane K. Helmer, Kyle T. Ganson, Alexander Testa, Jinbo He, Fiona C. Baker, Jason M. Lavender

**Affiliations:** 1 Department of Pediatrics, University of California, San Francisco, 550 16th Street, 4th Floor, Box 0503, San Francisco, CA 94143 USA; 2 Department of Psychology, Suffolk University, Boston, MA USA; 3 Factor-Inwentash Faculty of Social Work, University of Toronto, Toronto, Canada; 4 Department of Management, Policy and Community Health, University of Texas Health Science Center at Houston, Houston, TX USA; 5 Department of Biosciences and Bioinformatics, School of Science, Xi’an Jiaotong-Liverpool University, Suzhou, Jiangsu China; 6 Center for Health Sciences, SRI International, Menlo Park, CA USA; 7 Military Cardiovascular Outcomes Research Program (MiCOR), Department of Medicine, Uniformed Services University of the Health Sciences, Bethesda, MD USA; 8 The Metis Foundation, San Antonio, TX USA

**Keywords:** Body mass index, Weight, Eating disorders, Adolescence, Youth, Epidemiology

## Abstract

**Purpose:**

To investigate sex differences in prospective associations between eating disorder (ED) symptoms and changes in body mass index (BMI) percentile in early adolescence.

**Methods:**

This prospective study used survey data from 7111 participants aged 10–12 years at Year 1 from the Adolescent Brain Cognitive Development (ABCD) Study—a diverse, national sample of adolescents from 21 sites across the United States (US). Multivariable linear regression models were used to assess the prospective associations between ED symptoms at Year 1 and BMI percentile at Year 2, adjusting for covariates and BMI percentile at Year 1. Effect moderation was explored in sex-stratified models.

**Results:**

Sex modified the relationship between ED symptoms and changes in BMI percentile. Having binge eating symptoms (B = 3.65, 95% CI 1.80–5.51, *p* <0.001), distress related to binge eating (B = 2.79, 95% CI 0.05–5.53, *p* = 0.046), inappropriate compensatory behaviors (B = 6.39, 95% CI 2.16–10.62, *p* = 0.005), and fear of weight gain (B = 5.26, 95% CI 3.18–7.33, *p* < 0.001) at Year 1 were significantly associated with higher BMI percentile at Year 2 among males. In the overall sample, distress related to binge eating (B = 2.09, 95% CI 0.40–3.69, *p* = 0.017) was significantly associated with a higher BMI percentile 1 year later; the interaction by sex was not statistically significant.

**Discussion:**

ED symptoms were associated with higher BMI percentiles 1 year later in early adolescents, although sex-stratified findings revealed that many associations were significant only for males. Clinicians caring for early adolescents should consider evaluating for ED symptoms as potential risk factors for elevated weight.

**Level of evidence:**

Level III: Evidence obtained from well-designed cohort or case–control analytic studies.

**Supplementary Information:**

The online version contains supplementary material available at 10.1007/s40519-026-01824-w.

## Introduction

Obesity and eating disorders (EDs) are increasingly significant public health concerns due to their prevalence and negative impact on physical and psychological health [1, 2]. In the United States (US), an estimated 37% of children and adolescents aged 5–14 years have a body mass index (BMI) in the range of overweight (85th to 95th BMI percentile) or obesity (≥ 95th BMI percentile) [3]. The median age of ED onset is during early adolescence (ages 10–14) [4], a period of particular vulnerability due to the physical and socioemotional changes associated with pubertal development [5]. ED symptoms include a range of behavioral and cognitive-affective features, such as binge eating, inappropriate compensatory behaviors, self-worth tied to weight, and fear of weight gain [6, 7]. Moreover, ED symptoms have been associated with a variety of adverse outcomes, including lower health-related quality of life [8], poor dietary intake [9], alcohol and tobacco use [10], depression [11], and increased risk of developing a full-threshold ED [12].

Previous literature suggests an association between excess body weight and ED symptoms among youth [13, 14], and those with both ED symptoms and higher body weight may experience compounded impacts on health and psychosocial functioning [15]. Compared to young adults with an underweight (< 5th BMI percentile) or “normal” (5th to 85th BMI percentile) BMI, those categorized as having elevated BMI (e.g., overweight or obesity) are more likely to report disordered eating behaviors [16]. This seemingly increased risk may stem from various Western sociocultural factors, including media-driven ideal body standards, peer influences, and experiences of weight-related teasing and stigma [13, 17]. Evidence supports a bidirectional relationship between ED symptoms and higher BMI. For example, dieting and unhealthy weight-control methods during adolescence have been repeatedly shown to predict future increases in BMI [18–22]. Binge eating, which involves consuming unusually large amounts of food while experiencing distress, may also result in BMI increases during adolescence, though the evidence is limited [23–25]. Unhealthy weight control behaviors, including restriction, may promote binge eating, poor physical activity, and ultimately lead to weight gain [26], fostering a cycle of ED behaviors. Furthermore, sex differences in ED symptoms are notable. Societally, the feminine body ideal is thin and fit, leading to pressures for weight loss and associated behaviors among adolescent girls [27]. In contrast, the masculine body ideal is muscular and lean, with one-third of adolescent boys attempting to gain weight or muscle [27]. Males are also more likely to have atypical anorexia nervosa (i.e., symptoms of anorexia nervosa with recent significant weight loss, but absent current underweight status) and higher premorbid BMIs, which further speaks to sex-related differences in eating disorder pathology [28]. Therefore, there may be differential associations between ED symptoms and weight change, given distinctive psychological and sociocultural factors influencing these variables in males and females.

While prior cohort studies have focused on dieting, fewer have examined how subclinical ED symptoms—such as binge eating, associated distress, compensatory behaviors to lose weight, worry about weight gain, and self-worth tied to weight—may prospectively predict BMI during early adolescence. This study aimed to address that gap by exploring the prospective relationships between ED symptoms and BMI in a socio-demographically diverse, population-based sample of early adolescents in the US, as well as exploring potential sex-based differences in these associations.

## Methods

### Participants

This study used Year 1 and Year 2 data from the Adolescent Brain Cognitive Development (ABCD) Study, an ongoing longitudinal study of brain development and child health in the US. A total of 11,875 children (ages 9–10 years at baseline) were recruited across 21 sites. Additional details about the study’s design and methods are published elsewhere [29]. Due to the COVID-19 pandemic, the collection of anthropometric measurements was disrupted in Year 2, resulting in a reduced number with complete data. For the current study, we excluded 4767 participants who had missing data on BMI, ED symptoms, or covariates at Years 1 or 2. This resulted in a final sample of 7111 participants (see Additional file 1: Table S1 [online resource] for exclusion comparisons). Institutional Review Board (IRB) approval was received from the University of California, San Diego, and the respective IRBs of each study site, and all participants provided written assent and caregiver consent.

### Measures

#### ED symptoms (Year 1)

ED symptoms were measured via parent/caregiver reports, as these data were available at both Year 1 and Year 2, which was necessary for the study design. Assessment was conducted using the computerized Kiddie Schedule for Affective Disorders and Schizophrenia (KSADS-5), which applies Diagnostic and Statistical Manual of Mental Disorders, 5th Edition (DSM-5) criteria to classify child and adolescent mental health concerns [30]. Parents/caregivers provided information on the frequency, duration, and characteristics of five ED symptoms aligning with DSM-5 criteria for eating disorders, such as binge eating disorder and anorexia nervosa [31, 32]: (1) binge eating, (2) distress related to binge eating, (3) inappropriate compensatory behaviors to prevent weight gain, (4) worry about weight gain, and (5) self-worth tied to weight [28, 33, 34]. Binge eating symptoms were assessed with the following question: “In the past 2 weeks, how often has your child had eating binges, when he or she lost control of their eating and ate way more than he or she needed to because your child was unable to stop himself or herself from eating?” Distress related to binge eating, another feature of the DSM-5 diagnostic criteria for binge eating disorder [31], was assessed with the question, “How much discomfort or distress does binge eating cause your child?” Parents/caregivers responded to the question asking about compensatory behaviors their child may have used to prevent weight gain, including eating low-calorie foods, excessive exercise, vomiting, and using water pills, laxatives, or diet pills. Due to the low prevalence of these behaviors in the non-clinical sample of this study, a single compensatory behavior variable was used, reflecting the presence or absence of any of the behaviors. To assess worry about weight gain, parents/caregivers were asked: “In the past 2 weeks, how often has your child been preoccupied with gaining weight or worrying a lot about being fat?” Finally, self-worth tied to weight was assessed with this question: “Does your child feel like his or her self-worth is tied to his or her weight?” More details are available in Additional file 1: Table S2. We summed the five binary variables to create a cumulative ED-symptom score, categorized as 0, 1, or 2 or more symptoms. The “2 or more” category was used due to the low frequency of higher cumulative ED-symptom scores, which resulted in cell sizes too small for reliable analysis.

#### Body mass index (Year 2)

Body weight was measured using the Healthometer 844KL High-Capacity Digital Bathroom Scale (Health o Meter Professional Scales), while height was measured with a carpenter’s square steel tape measure. Both measurements were taken three times by trained research assistants, and the average was computed. BMI percentiles [35, 36] were obtained from height and weight according to guidelines from the Centers for Disease Control and Prevention (CDC), specific to age and sex [37]. Implausible BMI data from Year 1 and Year 2 were excluded based on established criteria: BMI z-scores ≤ -4 SDs or ≥ 8 SDs [38].

#### Covariates

Year 1 covariates were selected based on their theoretical relevance to the prospective relationship between ED symptoms and body weight [39], as well as prior evidence linking depression symptoms and sleep disturbance to both variables [40–43]. Covariates included age, sex, race/ethnicity, household income, highest parent education, study site, depression symptoms, and sleep disturbance.

Depression symptoms were measured using 13 questions from the DSM-5 Oriented Affective Problems Scale of the Child Behavior Checklist, (scale range 0–26) [44]. Sleep disturbance was measured using 26 Likert-type questions from the full Sleep Disturbance Scale for Children (score range: 26–130) [45].

In ancillary analyses, child-reported pubertal status was assessed using the Pubertal Development Scale. A mean pubertal status score was calculated, and a separate categorical pubertal status variable was defined with five categories: prepubertal, early pubertal, mid-pubertal, late pubertal, and post pubertal [46]. Due to small numbers in the post pubertal category, late and post pubertal stages were combined and analyzed as a single group (late/post pubertal).

### Statistical analysis

Descriptive statistics were conducted and stratified by sex, with differences assessed using Student’s *t*-tests and chi-square tests. Multivariable linear regression models were used to assess prospective associations of individual ED symptoms and the cumulative ED-symptom score at Year 1 with BMI percentile at Year 2, adjusting for age, sex, race, household income, highest parental education, study site, depression symptoms, sleep disturbance, and BMI percentile at Year 1. In ancillary analyses, models were further adjusted for pubertal status, modeled separately as a continuous variable and as a categorical variable in sensitivity analyses. Because pubertal status had missing data for 825 participants (11.6%), missing values were imputed using multiple imputation by chained equations (Additional file 1: Table S3).

We conducted sensitivity analyses assessing prospective associations of ED symptoms at Year 1 with binary BMI percentile ≥ 85th percentile at Year 2 using multivariable logistic regression, adjusting for the same covariates (Additional file 1: Table S4). We assessed BMI percentile at Year 1 in association with BMI percentile at Year 2 (Additional file 1: Table S5). We also examined cross-sectional associations between ED symptoms and BMI percentile at Year 1 (Additional file 1: Table S6).

We also examined prospective associations of BMI at Year 1 with ED symptoms at Year 2 using logistic regression (Additional file 1: Table S7). For these analyses, BMI z-scores were used to improve the interpretability of effect estimates, given that BMI percentile is bounded and small percentile changes may be difficult to interpret. Analyses were repeated using BMI percentile to assess the robustness of findings.

We tested whether there was an interaction between ED symptoms and sex, and between BMI and sex, and then performed sex-stratified models to explore effect modification. A *p*-value of < 0.05 was considered to indicate statistical significance. Sample weights derived from the American Community Survey of the US Census were applied.

## Results

### Participant characteristics

A total of 7111 early adolescents aged 10–12 years at Year 1 were included in this study. Nearly half (47.4%) of the participants were female, and 42.3% were non-White. Compared to females, males had a higher prevalence of binge eating symptoms and a lower prevalence of worry about weight gain (Table 1).

**Table 1:** Sociodemographic and health characteristics of participants in the Adolescent Brain Cognitive Development (ABCD) Study at year 1 (n = 7111)

Sociodemographic characteristics	All	Female	Male	*p*-value
	n = 7111	n = 3373	n = 3738	
	Mean (SD)/n (%)	Mean (SD)/n (%)	Mean (SD)/n (%)	
Age (years), mean (SD)	11.0 (0.6)	10.9 (0.6)	11.0 (0.6)	0.066
Parent’s highest education, n (%)				
High school education or less	616 (8.7%)	299 (8.9%)	317 (8.5%)	0.565
College education or more	6495 (91.3%)	3074 (91.1%)	3421 (91.5%)	
Household income, n (%)				
Less than $25,000	880 (12.4%)	412 (12.2%)	468 (12.5%)	0.173
$25,000 through $49,999	988 (13.9%)	474 (14.1%)	514 (13.8%)	
$50,000 through $74,999	960 (13.5%)	435 (12.9%)	525 (14.0%)	
$75,000 through $99,999	1049 (14.8%)	535 (15.9%)	514 (13.8%)	
$100,000 through $199,999	2342 (32.9%)	1097 (32.5%)	1245 (33.3%)	
$200,000 and greater	892 (12.5%)	420 (12.5%)	472 (12.6%)	
Race, n (%)				
Asian	417 (5.9%)	208 (6.2%)	209 (5.6%)	0.530
Black	1140 (16.0%)	565 (16.8%)	575 (15.4%)	
Latino/Hispanic	1114 (15.7%)	516 (15.3%)	598 (16.0%)	
Native American	246 (3.5%)	116 (3.4%)	130 (3.5%)	
White	4104 (57.7%)	1927 (57.1%)	2177 (58.2%)	
Other	90 (1.3%)	41 (1.2%)	49 (1.3%)	
Depression symptoms (raw score), mean (SD)	1.4 (2.1)	1.3 (2.1)	1.5 (2.2)	** < 0.001**
Sleep disturbance, mean (SD)	36.5 (7.9)	36.4 (7.8)	36.6 (8.0)	0.349
BMI percentile at Year 1, mean (SD) [range]	61.5 (30.8) [0–99.9]	60.2 (31.1) [0–99.9]	62.8 (30.5) [0–99.8]	** < 0.001**
BMI percentile at Year 2, mean (SD) [range]	62.1 (30.7) [0–99.8]	65.0 (30.6) [0–99.8]	59.5 (30.5) [0–99.8]	** < 0.001**
Binge eating symptoms, n (%)				
No	6725 (95.5%)	3198 (96.1%)	3527 (95.0%)	**0.029**
Yes	315 (4.5%)	130 (3.9%)	185 (5.0%)	
Distress related to binge eating, n (%)				
No	6900 (97.0%)	3279 (97.2%)	3621 (96.9%)	0.394
Yes	211 (3.0%)	94 (2.8%)	117 (3.1%)	
Inappropriate compensatory behaviors, n (%)				
No	6885 (96.8%)	3269 (96.9%)	3616 (96.7%)	0.665
Yes	226 (3.2%)	104 (3.1%)	122 (3.3%)	
Worry about weight gain, n (%)				
No	7069 (99.4%)	3346 (99.2%)	3723 (99.6%)	**0.028**
Yes	42 (0.6%)	27 (0.8%)	15 (0.4%)	
Self-worth tied to weight, n (%)				
No	7015 (98.6%)	3327 (98.6%)	3688 (98.7%)	0.924
Yes	96 (1.4%)	46 (1.4%)	50 (1.3%)	
Cumulative score				
0	6663 (93.7%)	3169 (94.0%)	3494 (93.5%)	0.531
1	352 (5.0%)	157 (4.7%)	195 (5.2%)	
2+	96 (1.4%)	47 (1.4%)	157 (1.3%)	
Pubertal status				** < 0.001**
Prepubertal	2238 (20.9%)	663 (13.6%)	1575 (27.4%)	
Early pubertal	3414 (33.4%)	942 (19.3%)	2471 (46.0%)	
Mid-pubertal	3670 (37.1%)	2432 (51.6%)	1238 (24.2%)	
Late/post pubertal	732 (8.6%)	627 (15.5%)	105 (2.4%)	

Sampling weights were applied to yield estimates based on the American Community Survey from the US Census. Bold values indicate statistical significance p<0.05

### Prospective associations of ED symptoms and BMI

Table 2 shows adjusted associations between ED symptoms at Year 1 and BMI percentile at Year 2. The presence (versus absence) of binge eating symptoms (B = 2.15, 95% CI 0.97–3.33) and distress related to binge eating (B = 2.09, 95% CI 0.40–3.69) were associated with higher BMI percentile at Year 2. Presence of inappropriate compensatory behaviors (B = 3.20, 95% CI 0.83–5.58) and worry about weight gain (B = 3.42, 95% CI 1.05–5.80) was also associated with higher BMI percentile at Year 2. For the cumulative ED-symptom score, participants with one symptom (B = 3.39, 95% CI 1.98–4.80) or with two or more symptoms (B = 2.41, 95% CI 0.90–3.92) at Year 1 had a higher BMI percentile at Year 2 compared to those without symptoms. Significant interactions by sex were observed for binge eating symptoms, inappropriate compensatory behaviors, worry about weight gain, and the cumulative ED-symptom score (Table 2). In sex-stratified models, associations were significant among males but not females. Among males, having binge eating symptoms (B = 3.65, 95% CI 1.80–5.51), distress related to binge eating (B = 2.79, 95% CI 0.05–5.53), inappropriate compensatory behaviors (B = 6.39, 95% CI 2.16–10.62), and fear of weight gain (B = 5.26, 95% CI 3.18–7.33) at Year 1 were significantly associated with higher BMI percentile at Year 2.

**Table 2:** Prospective associations between eating disorder symptoms at year 1 and body mass index (BMI) percentile at year 2 in the Adolescent Brain Cognitive Development (ABCD) Study

	Adjusted		*p*-interaction	Female		Male	
	Coefficient (95% CI)	*p*		Coefficient (95% CI)	*p*	Coefficient (95% CI)	*p*
Binge eating symptoms	**2.15 (0.97, 3.33)**	**0.001**	** < 0.001**	0.32 (− 1.08, 1.74)	0.635	**3.65 (1.80, 5.51)**	** < 0.001**
Distress related to binge eating	**2.09 (0.40, 3.69)**	**0.017**	0.113	0.56 (− 1.07, 2.21)	0.482	**2.79 (0.05, 5.53)**	**0.046**
Inappropriate compensatory behaviors	**3.20 (0.83, 5.58)**	**0.011**	**0.003**	− 0.41 (− 2.21, 1.39)	0.640	**6.39 (2.16, 10.62)**	**0.005**
Worry about weight gain	**3.42 (1.05, 5.80)**	**0.007**	**0.046**	2.46 (− 0.98, 5.92)	0.152	**5.26 (3.18, 7.33)**	** < 0.001**
Self-worth tied to weight	0.71 (− 0.50, 1.93)	0.238	0.179	− 0.38 (− 2.59, 1.83)	0.723	1.79 (− 0.94, 4.52)	0.187
Cumulative score							
0	Ref.	Ref.	** < 0.001**	Ref.	Ref.	Ref.	Ref.
1	**3.39 (1.98, 4.80)**	** < 0.001**		0.76 (− 0.89, 2.41)	0.349	**5.70 (2.73, 8.69)**	**0.001**
2+	**2.41 (0.90, 3.92)**	**0.003**		0.60 (− 0.88, 2.08)	0.406	**3.75 (1.48, 6.03)**	**0.003**

Models adjusted for study site, race/ethnicity, and Year 1 measures of age, household income, parental education, depression symptoms, sleep disturbance, and BMI percentile. Sample weights derived from the American Community Survey of the US Census were applied. Bold values indicate statistical significance p<0.05

Results from models additionally adjusted for pubertal status as a categorical variable are shown in Additional file 1: Table S3. Estimates were largely unchanged when pubertal status was modeled as either continuous or categorical; however, the association between self-worth tied to weight and BMI percentile was attenuated and no longer statistically significant in the full sample and among males.

In sensitivity analyses, binge eating symptoms, distress related to binge eating, worry about weight gain, and a higher cumulative ED-symptom score were prospectively associated with a BMI percentile of ≥ 85th (Additional file 1: Table S4). Year 1 BMI percentile was associated with Year 2 BMI percentile for all ED symptom models (Additional file 1: Table S5). Cross-sectionally, all ED symptoms were associated with BMI percentile at Year 1 (Additional file 1: Table S6). With ancillary analyses, a higher BMI z-score at Year 1 was associated with greater odds of ED symptoms, with effect modification of the BMI association by sex observed for self-worth tied to weight only and higher cumulative symptom category at Year 2, with limited evidence of effect modification by sex (Additional file 1: Table S7).

## Discussion

Findings indicate that having certain ED symptoms was prospectively associated with a higher BMI percentile one year later in early adolescents. Specifically, binge eating symptoms, distress related to binge eating, compensatory behaviors, and worry about weight gain each predicted an increase in BMI percentile in the following year, which was magnified when multiple ED symptoms were present. Notably, associations were more pronounced in males, suggesting potential sex differences in vulnerability to weight gain in the presence of ED symptoms. Screening and targeted prevention may therefore be important for improving the long-term health outcomes of early adolescents.

The transdiagnostic model of eating disorders that underlies enhanced cognitive behavioral therapy (CBT-E) provides insights into the cyclical nature of ED symptoms [47]. Per this model, the overvaluation of eating, shape, and weight promotes restrictive eating and weight control behaviors. Such behaviors increase risk for loss of control while overeating (i.e., binge eating), which in turn prompts compensatory behaviors and restriction, perpetuating a cycle that may ultimately lead to escalating symptoms and weight gain [47].

We found that binge eating symptoms predicted a 2.15-unit higher BMI percentile at one-year follow-up among early adolescents, and distress associated with binge eating also predicted a 2.09-unit higher BMI percentile. This aligns with prior cross-sectional and longitudinal studies across 9–12- and 13-year-old populations in Finland and the UK, which have shown associations between binge eating and elevated BMI *z*-scores [23, 24]. Among young adults, those with binge eating episodes had 74% higher odds of developing overweight or obesity in the following 6–10 years [25].

Inappropriate compensatory behaviors to prevent weight gain were associated with a 3.20-unit higher BMI percentile one year later, and a 6.39-unit higher BMI percentile for males. This finding is consistent with prior research, including a midwestern US study linking unhealthy weight-control behaviors to higher BMI over time among adolescents [18]. A prospective cohort study of adolescents also found that dieters gained more weight than non-dieters [21], and other research found that females who dieted during adolescence had larger gains in BMI during the transition to young adulthood [22]. Similarly, female adolescents in a community-based study demonstrated increases in relative body weight associated with weight-reduction efforts [19]. Our study highlights a stronger association in males than in females.

Fear of weight gain, a key cognitive-affective symptom theorized to maintain EDs [48], was associated with a 3.42-unit higher BMI percentile one year later. This is consistent with previous findings that identified cognitive symptoms, such as weight and shape concerns, as predictors of BMI increases in early adolescent girls [24]. In contrast, no significant association was observed between weight-based self-worth and an increase in BMI percentile at one-year follow-up. Alongside the notable link between distress over binge eating and weight gain, these results may highlight the importance of affective ED symptoms, in addition to cognitive ED symptoms, and their role in adolescent weight gain.

Despite the higher overall prevalence of ED symptoms typically observed in female versus male youth [49], our study found that binge eating symptoms were more common in males (5.0%) than in females (3.9%). Male youth may experience binge eating in relation to attempts to achieve the simultaneously muscular (i.e., “bigger”, greater size/bulk) and lean (i.e., low body fat) Western male body ideal. This notion is also consistent with the present finding that in addition to binge eating symptoms and distress related to binge eating, compensatory behaviors, and worry about weight gain exhibited larger associations with BMI percentile increases in males compared to females. Although compensatory behaviors may be intended to offset weight gain [50], they commonly have paradoxical effects, with persistent patterns of binge eating and compensatory behaviors increasing the risk of weight cycling and long-term weight gain. These findings may not have been as pronounced in females as there is greater variability in BMI percentile among males [51], and the difference in Western body ideals may have led to the difference in trajectories, as the feminine body ideal within Western societies is thin and fit, leading to pressures for weight loss among adolescent girls [27]. Conversely, the masculine body ideal is muscular and lean, leading to pressures for building muscle and associated behaviors among adolescent boys [27].

These findings have associated clinical and public health implications. ED symptoms tied to elevated weight may be a culprit in many poor health outcomes [52]. With participants across the weight spectrum, our study reinforces that ED screening and prevention efforts should not be limited to youth with low weight status [53]. Therefore, youth who present with higher weight status should be evaluated for ED symptoms, particularly given the link between the two during early adolescence [13]. As the traditional Western male body image ideal is to be “bigger” (have more lean muscle), males who are engaging in disordered eating behaviors may be trying to gain weight and muscle while limiting body fat. Therefore, as males with higher BMI percentiles are at risk for several ED symptoms, it is important to design tailored interventions to address Western gender sociocultural norms and prevention efforts for this population. These efforts should include targeting males specifically, considering ED risk in obesity care for boys and men, and counseling boys and men with higher weight status about ED risk and symptoms, as Western gender norms do not typically recognize that males pursuing the body ideal may also be at risk for eating disorders [54, 55].

### Strengths and limitations

This study had several limitations. Although our analysis was prospective, the observational design restricts causal inference, and the relationship between ED symptoms and BMI percentile could be bidirectional. Further, incremental changes in 2–5 units of BMI percentile, in reality, correspond with a minor change in actual weight in pounds. However, prior studies have found that girls with higher BMI at ages 9 and 11 are more likely to have greater waist-to-height ratios and greater than expected changes in BMI at age 18, suggesting that even small increases in BMI during early adolescence may contribute to higher BMI trajectories that persist across the life course [56]. Additionally, parent reports were used due to data availability at both Year 1 and 2, which was necessary for the prospective design. Adolescent self-reports were not available at both time points, precluding their inclusion. Although previous research using Year 2 data from the ABCD Study has identified discrepancies between parent and adolescent reports of ED symptoms [58], we were unable to examine informant differences in the current study due to the absence of adolescent self-report data at Year 1 and the need to preserve proper temporal ordering. Certain ED symptoms, such as binge eating or compensatory behaviors, may be more commonly reported by adolescents due to their private or embarrassing nature, while others may be more accurately observed by parents, potentially because adolescents may lack insight or deny the issue [59–61]. Additionally, behaviors such as overeating without loss of control, may also be misclassified as binge eating given the difficulty in assessing adolescents’ subjective sense of loss of control when using parent-reported measures. This is particularly challenging given the normal changes to adolescent eating behaviors during puberty, which often includes eating more overall. Therefore, future studies should consider both parent and adolescent reports of ED symptoms. Additionally, missing data may have influenced our findings, as excluded participants tended to be slightly younger, have parents with lower educational attainment, have lower household income, and identify as racial/ethnic minorities (Additional file 1: Table S1). Therefore, our results may not be fully generalizable, and given that these characteristics are associated with higher BMI and obesity prevalence [62], our results may underestimate the true magnitude of associations.

Despite these limitations, key strengths include a large, diverse, national sample of early adolescents and a prospective design with a one-year follow-up period. We controlled for baseline BMI to isolate the effect of ED symptoms and included adolescents regardless of initial BMI status. Additionally, our analysis identified male adolescents as particularly vulnerable to increased BMI percentile associated with ED symptoms.

## Conclusion

Our study found that ED symptoms were prospectively associated with a higher BMI percentile one year later in a population of early adolescents, with stronger associations among males. Key findings contribute to the growing evidence linking unhealthy weight control behaviors and binge eating with subsequent weight gain in adolescents. Future research should continue to study these relationships longitudinally and incorporate adolescent self-reports to highlight subjective experiences.

### What is already known about this subject?

Higher weight status and ED symptoms affect many children and adolescents and have adverse effects on physical and mental health. Adolescents with excess body weight are more likely to engage in disordered eating—this relationship is likely bidirectional. Existing studies have shown that various unhealthy weight control behaviors (i.e., dieting) and disordered eating behaviors predict weight gain during adolescence. However, there is limited data on how ED symptoms relate to changes in BMI among early adolescents, and how these associations may differ by sex.

### What does this study add?

This study provides evidence that subclinical ED symptoms (binge eating symptoms, distress related to binge eating, inappropriate compensatory behaviors, and fear of weight gain) are associated with a higher BMI percentile one year later among a diverse sample of early adolescents. Sex modified this relationship. Early adolescent males, compared to females, demonstrated significant and more pronounced increases in BMI percentile for many ED symptoms.

## Supplementary Information

Below is the link to the electronic supplementary material.

Supplementary file 1 (DOCX 38 KB)

## Data Availability

Data used in the preparation of this article were obtained from the ABCD Study (https://abcdstudy.org), held in the NIH Brain Development Cohorts (NBDC) Portal.
