# Supplementary material for: Eating disorder symptoms are prospectively associated with higher BMI percentile in male early adolescents
Source: Eat Weight Disord. 2026 Feb 16;31(1):21. doi: 10.1007/s40519-026-01824-w (PMC13013186; doi:10.1007/s40519-026-01824-w)
Supplement: Supplementary file 1 — (DOCX 38 KB) [file 40519_2026_1824_MOESM1_ESM.docx]

**Supplementary Information**

**Eating disorder symptoms are prospectively associated with higher BMI percentile in male early adolescents**

Jason M. Nagata, MD, MSc, Abubakr A. Al-Shoaibi, PhD, Shayna Weinstein, MPH, Zain Memon, Elizabeth J. Li, MPH, Wesley R. Barnhart, PhD, Christiane K. Helmer, MPH, Kyle T. Ganson, PhD, Alexander Testa, PhD, Jinbo He, PhD, Fiona C. Baker, PhD, Jason M. Lavender, PhD

**Corresponding Author:**

Jason M. Nagata, M.D., M.Sc.

E-mail: [jason.nagata@ucsf.edu](mailto:jason.nagata@ucsf.edu)

| **Sociodemographic characteristics** | Included  n=7111 | Excluded  n=3738 |  |
| --- | --- | --- | --- |
|  | **Mean (SD) / n (%)** | **Mean (SD) / n (%)** | **p-value** |
| Age (years), mean (SD) | 11.0 (0.7) | 10.9 (0.6) | **<0.001** |
| Sex, n (%) |  |  |  |
| Female | 3,373 (47.4%) | 2,307 (48.4%) | 0.294 |
| Male | 3,738 (52.6%) | 2,458 (51.6%) |  |
| Parent's highest education, n (%) |  |  |  |
| High school education or less | 616 (8.7%) | 762 (17.8%) | **<0.001** |
| College education or more | 6,495 (91.3%) | 3,523 (82.2%) |  |
| Household income, n (%) |  |  |  |
| Less than $25,000 | 880 (12.4%) | 919 (20.8%) | **<0.001** |
| $25,000 through $49,999 | 988 (13.9%) | 677 (15.3%) |  |
| $50,000 through $74,999 | 960 (13.5%) | 559 (12.6%) |  |
| $75,000 through $99,999 | 1,049 (14.8%) | 521 (11.8%) |  |
| $100,000 through $199,999 | 2,342 (32.9%) | 1,228 (27.7%) |  |
| $200,000 and greater | 892 (12.5%) | 524 (11.8%) |  |
| Race, n (%) |  |  |  |
| Asian | 417 (5.9%) | 292 (6.1%) | **<0.001** |
| Black | 1,140 (16.0%) | 1,252 (26.3%) |  |
| Latino / Hispanic | 1,114 (15.7%) | 914 (19.2%) |  |
| Native American | 246 (3.5%) | 164 (3.4%) |  |
| White | 4,104 (57.7%) | 2,064 (43.3%) |  |
| Other | 90 (1.3%) | 81 (1.7%) |  |

Supplementary Table 1. Comparison of characteristics between participants with and without missing data at Year 1.

Sampling weights were applied to yield estimates based on the American Community Survey from the US Census.

SD = standard deviation.

| Supplementary Table 2. KSADS-5 Assessment of Eating Disorder Symptoms in the Adolescent Brain Cognitive Development (ABCD) Study | | |
| --- | --- | --- |
| **Symptom** | **Question** | **Comments** |
| Binge Eating | In the past two weeks, how often has your child had eating binges, when he or she lost control of his or her eating and ate way more than he or needed, because your child was unable to stop yourself from eating? | Parents/caregivers who responded “yes” on behalf of their child were coded as binge eating. |
| Distress Related to Binge Eating | How much discomfort or distress does binge eating cause your child? | Parents/caregivers were asked to select a response ranging from 0 to 10. The Kiddie Schedule for Affective Disorders and Schizophrenia (KSADS-5) assessment used a response of 3 or higher as the cutoff to indicate having distress with binge eating. |
| Inappropriate Compensatory Behaviors to Prevent Weight Gain | Please note below all the different methods that your child has used to control his or her weight. | Parents/caregivers were asked to select from the following behaviors: diet pills, laxatives, water pills, throwing up, exercising a lot, only eating foods or drinks with minimal calories (e.g., carrots, celery, zero calorie drinks), or other (fill in). Those who selected at least one response option on behalf of their child were coded as engaging in inappropriate behaviors to prevent weight gain. |
| Worry About Weight Gain | In the past two weeks, how often has your child been preoccupied with his or her weight or worrying a lot about being fat? | Parents/caregivers selected responses as follows: Not at all, rarely, several days, more than half the days, and nearly every day. Those who responded “nearly every day” on behalf of their child were coded as worrying about weight gain. |
| Self-Worth Tied to Weight | Do you feel like your child’s self-worth is tied to his or her weight? | Parents/caregivers who responded “yes” on behalf of their child were coded as feeling self-worth tied to weight. |

Supplementary Table 3. Prospective associations between eating disorder symptoms at Year 1 and BMI percentile at Year 2 in the Adolescent Brain Cognitive Development (ABCD) Study

|  | Adjusted | | | Female | | Male | |
| --- | --- | --- | --- | --- | --- | --- | --- |
|  | Coefficient (95% CI) | p | p-interaction | Coefficient (95% CI) | p | Coefficient (95% CI) | p |
| Binge eating symptoms | **2.17 (0.88, 3.46)** | **0.001** | **0.001** | 0.35 (-1.15, 1.86) | 0.644 | **3.64 (1.53, 5.75)** | **0.001** |
| Distress associated with binge eating | 2.06 (-0.17, 4.29) | 0.070 | 0.159 | 0.54 (-1.97, 3.04) | 0.676 | 2.81 (-0.77, 6.40) | 0.124 |
| Inappropriate compensatory behaviors | **3.24 (0.94, 5.54)** | **0.006** | 0.406 | -0.52 (-2.31, 1.27) | 0.569 | **6.52 (2.64, 10.40)** | **0.001** |
| Worry about weight gain | **3.23(0.72, 5.74)** | **0.011** | 0.062 | 2.15 (-1.27, 5.57) | 0.152 | **5.34 (3.23, 7.44)** | **<0.001** |
| Self-worth tied to weight | 0.59 (-0.50, 2.61) | 0.563 | 0.521 | -0.50 (-2.98, 1.98) | 0.691 | 1.77 (-1.45, 5.00) | 0.281 |
| Cumulative score |  |  |  |  |  |  |  |
| 0 | Ref | Ref | **0.004** | Ref | Ref | Ref | Ref |
| 1 | **3.48 (1.53, 5.43)** | **<0.001** |  | 0.92 (-0.91, 2.74) | 0.324 | **5.31 (2.07, 8.53)** | **0.001** |
| 2+ | 1.61 (-0.30, 3.57) | 0.099 |  | -0.10 (-2.45, 2.24) | 0.930 | **3.47 (0.56, 6.39)** | **0.019** |

Model adjusted for study site, race/ethnicity, and Year 1 measures of age, household income, parental education, sleep disturbance, BMI percentile, depression symptoms, and puberty status (categorical). Sampling weights were applied to yield estimates based on the American Community Survey from the US Census.

| Supplementary Table 4. Prospective associations between eating disorder symptoms at Year 1 and body mass index (BMI) percentile ≥85^th^ at Year 2 in the Adolescent Brain Cognitive Development (ABCD) Study | | |
| --- | --- | --- |
|  | Adjusted OR (95% CI) | p |
| Binge eating symptoms | **2.15 (1.59, 2.92)** | **<0.001** |
| Distress associated with binge eating | **1.92 (1.35, 2.75)** | **0.001** |
| Inappropriate compensatory behaviors | 1.57 (0.94, 2.61) | 0.082 |
| Worry about weight gain | **9.65 (2.43, 38.38)** | **0.003** |
| Self-worth tied to weight | 1.21 (0.61, 2.44) | 0.567 |
| Cumulative score |  |  |
| 0 | Ref | Ref |
| 1 | **1.85 (1.45, 1.37)** | **<0.001** |
| 2+ | **2.41 (1.78, 3.51)** | **<0.001** |
| Models adjusted for study site, race/ethnicity, and Year 1 measures of age, household income, parental education, depression symptoms, sleep disturbance, and BMI percentile. Sampling weights were applied to yield estimates based on the American Community Survey from the US Census.  Ref = BMI percentile <85^th^ | | |

Supplementary Table 5. Prospective associations between BMI percentile at Year 1 and BMI percentile at Year 2 for each eating disorder symptom model in the Adolescent Brain Cognitive Development (ABCD) Study

|  | All | | Female | | Male | |
| --- | --- | --- | --- | --- | --- | --- |
|  | Coefficient (95% CI) | p | Coefficient (95% CI) | p | Coefficient (95% CI) | p |
| **Binge-eating symptoms model** |  |  |  |  |  |  |
| BMI percentile at Year 1 | **0.86 (0.84, 0.89)** | **<0.001** | **0.84 (0.81, 0.87)** | **<0.001** | **0.87 (0.85, 0.91)** | **<0.001** |
| **Distress associated with binge eating model** |  |  |  |  |  |  |
| BMI percentile at Year 1 | **0.87 (0.84, 0.89)** | **<0.001** | **0.84 (0.81, 0.87)** | **<0.001** | **0.88 (0.86, 0.91)** | **<0.001** |
| **Inappropriate compensatory behaviors model** |  |  |  |  |  |  |
| BMI percentile at Year 1 | **0.87 (0.84, 0.88)** | **<0.001** | **0.84 (0.80, 0.87)** | **<0.001** | **0.88 (0.85, 0.91)** | **<0.001** |
| **Worry about weight gain model** |  |  |  |  |  |  |
| BMI percentile at Year 1 | **0.87 (0.84, 0.88)** | **<0.001** | **0.84 (0.80, 0.87)** | **<0.001** | **0.89 (0.86, 0.91)** | **<0.001** |
| **Self-worth tied to weight model** |  |  |  |  |  |  |
| BMI percentile at Year 1 | **0.87 (0.84, 0.89)** | **<0.001** | **0.84 (0.81, 0.87)** | **<0.001** | **0.89 (0.86, 0.91)** | **<0.001** |
| **Cumulative score model** |  |  |  |  |  |  |
| BMI percentile at Year 1 | **0.86 (0.84, 0.89)** | **<0.001** | **0.84 (0.80, 0.87)** | **<0.001** | **0.88 (0.85, 0.91)** | **<0.001** |

Model adjusted for study site, race/ethnicity, and Year 1 measures of age, household income, parental education, depression symptoms, sleep disturbance, and respective eating disorder symptom. Sampling weights were applied to yield estimates based on the American Community Survey from the US Census.

Supplementary Table 6. Cross-sectional associations between eating disorder symptoms and BMI percentile at Year 1 in the Adolescent Brain Cognitive Development (ABCD) Study

|  | All | |  | Female | | Male | |
| --- | --- | --- | --- | --- | --- | --- | --- |
|  | Coefficient (95% CI) | p | p-interaction | Coefficient (95% CI) | p | Coefficient (95% CI) | p |
| Binge-eating symptoms | **24.10 (20.68, 27.50)** | **<0.001** | 0.840 | **23.16 (19.57, 26.74)** | **<0.001** | **24.36 (19.50, 29.21)** | **<0.001** |
| Distress associated with binge eating | **21.97 (17.44, 26.52)** | **<0.001** | 0.930 | **21.38 (15.46, 27.31)** | **<0.001** | **22.38 (15.83, 28.93)** | **<0.001** |
| Inappropriate compensatory behaviors | **13.81 (10.65, 16.97)** | **<0.001** | 0.320 | **14.83 (11.26, 18.41)** | **<0.001** | **12.42 (6.69, 18.14)** | **<0.001** |
| Worry about weight gain | **24.86 (18.81, 30.91)** | **<0.001** | 0.100 | **21.59 (12.12, 31.05)** | **<0.001** | **29.69 (26.57, 32.80)** | **<0.001** |
| Self-worth tied to weight | **15.19 (7.63 22.75)** | **<0.001** | **0.021** | **19.63 (14.30, 24.95)** | **<0.001** | 8.53 (-2.67, 19.72) | 0.128 |
| Cumulative score |  |  |  |  |  |  |  |
| 0 | Ref | Ref | 0.534 | Ref | Ref | Ref | Ref |
| 1 | **16.25 (13.08, 19.41)** | **<0.001** |  | **17.15 (12.03, 22.27)** | **<0.001** | **15.22 (10.96, 19.49)** | **<0.001** |
| 2+ | **22.24 (17.29, 27.20)** | **<0.001** |  | **22.38 (17.24, 27.51)** | **<0.001** | **21.73 (12.53, 30.94)** | **<0.001** |
| Models adjusted for study site, race/ethnicity, and Year 1 measures of age, household income, parental education, depression symptoms, and sleep disturbance. Sampling weights were applied to yield estimates based on the American Community Survey from the US Census. | | | | | | | |

Supplementary Table 7. Prospective associations between BMI z-score at Year 1 and eating disorder (ED) symptoms at Year 2 in the Adolescent Brain Cognitive Development (ABCD) Study

|  | Adjusted | |  | Female | | Male | |
| --- | --- | --- | --- | --- | --- | --- | --- |
|  | OR (95% CI) | p | p-interaction | OR (95% CI) | p | OR (95% CI) | p |
| Binge eating symptoms | **1.67 (1.52, 1.84)** | **<0.001** | 0.672 | **1.61 (1.28, 2.02)** | **<0.001** | **1.74 (1.42, 2.13)** | **<0.001** |
| Self-worth tied to weight | **1.45 (1.08, 1.95)** | **0.015** | **0.045** | 1.14 (0.80, 1.61) | 0.674 | **1.86 (1.27, 2.72)** | **0.003** |
| Distress associated with binge eating | **2.21 (1.85, 2.64)** | **<0.001** | 0.664 | **2.33 (1.76, 3.10)** | **<0.001** | **2.15 (1.52, 3.04)** | **<0.001** |
| Inappropriate compensatory behaviors | **1.52 (1.24, 1.87)** | **<0.001** | 0.143 | **1.38 (1.02, 1.86)** | **0.038** | **1.76 (1.31, 2.36)** | **0.001** |
| Fear of gaining weight | **2.03 (1.55, 2.67)** | **<0.001** | 0.941 | **1.96 (1.28, 3.01)** | **0.004** | **2.28 (1.47, 3.54)** | **0.001** |
| ED cumulative score | **1.83 (1.66, 2.04)** | **<0.001** | 0.091 | **1.67 (1.40, 2.00)** | **<0.001** | **2.03 (1.72, 2.39)** | **<0.001** |

Multivariable logistic regression (for individual ED symptoms) and multinomial logistic regression (for ED cumulative score) adjusted for study site, race/ethnicity, and Year 1 measures of age, household income, parental education, sleep disturbance, and depression symptoms. Sampling weights were applied to yield estimates based on the American Community Survey from the US Census.
